# Rapid killing of *Capnocytophaga canimorsus* and *Capnocytophaga cynodegmi* by human whole blood and serum is mediated via the complement system

**DOI:** 10.1186/s40064-015-1308-9

**Published:** 2015-09-17

**Authors:** Salah Zangenah, Peter Bergman

**Affiliations:** Div of Clinical Microbiology, F68, Department of Laboratory Medicine, Karolinska Institutet and Karolinska University Hospital, Huddinge, Stockholm, Sweden; Department of Medicine, Center for Infectious Medicine (CIM), Karolinska Institutet and Karolinska University Hospital, Huddinge, Stockholm, Sweden

**Keywords:** *Capnocytophaga canimorsus*, *Capnocytophaga cynodegmi*, Serum killing assay, Whole blood killing assay, Classical pathway, Alternative pathway, Complement system, Mannose binding lectin deficiency

## Abstract

**Purpose:**

*Capnocytophaga canimorsus* (*Cani*) and *Capnocytophaga cynodegmi* (*Cyno*) are found in the oral cavities of dogs and cats. They can be transmitted to humans via licks or bites and cause wound infections as well as severe systemic infections. *Cani* is considered to be more pathogenic than *Cyno*, but the pathophysiological mechanisms are not elucidated. *Cani* has been suggested to be resistant to serum bactericidal effects. Thus, we hypothesized that the more invasive *Cani* would exhibit a higher degree of serum-resistance than the less pathogenic *Cyno.*

**Methods:**

Whole blood and serum bactericidal assays were performed against *Cani*- (n = 8) and *Cyno*-strains (n = 15) isolated from blood and wound-specimens, respectively. Analysis of complement-function was performed by heat-inactivation, EGTA-treatment and by using C1q-depleted serum. Serum and whole blood were collected from healthy individuals and from patients (n = 3) with a history of sepsis caused by *Cani*.

**Results:**

Both *Cani* and *Cyno* were equally susceptible to human whole blood and serum. *Cani* was preferentially killed by the classical pathway of the complement-system whereas *Cyno* was killed by a partly different mechanism. Serum from 2/3 *Cani*-infected patients were deficient in MBL-activity but still exhibited the same killing effect as control sera.

**Conclusion:**

Both *Cani* and *Cyno* were readily killed by human whole blood and serum in a complement-dependent way. Thus, it is not likely that serum bactericidal capacity is the key determinant for the clinical outcome in *Cani* or *Cyno*-infections.

## Background

The gram negative bacteria *Capnocytophaga canimorsus* (*Cani*) and *Capnocytophaga cynodegmi* (*Cyno*) constitute a significant part of the oral flora of dogs and cats (Suzuki et al. 2010). *Cani* was first described in 1976 and originally named dysgonic fermenter 2 (DF-2) (Bobo and Newton 1976). In the same report a ‘DF-2 like’ bacteria, mainly associated with wound infections, was described. Later, DF2 was named *Capnocytophaga canimorsus* (‘canimorsus’ is latin for ‘dogbite’) and “DF-2 like” *Capnocytophaga cynodegmi* (‘cynodegmi’ is greek for ‘dogbite’) (Brenner et al. 1989). *Cani* can cause severe infections, including sepsis, meningitis or endocarditis, after contact with dogs or cats (Oehler et al. 2009). The clinical picture is characterized by a rapid onset a few days after animal contact, often with a fulminant course (Oehler et al. 2009) and sometimes with neurological sequelae (Gasch et al. 2009). In contrast to *Cani*, *Cyno* is not as well described, but the general notion is that *Cyno* mainly is found in wound infections and rarely cause invasive infections. Most *Cani*-strains are susceptible to empirical treatment with ampicillin together with clavulanic acid (Oehler et al. 2009; Butler 2015).

The diagnostic microbiology is notoriously difficult, mainly due to the fastidious nature and slow growth on standard agar media. *Cani* and *Cyno* are facultative anaerobic bacteria that grow best on blood and hematin agar plates. In addition, the presence of 5–10 % CO_2_ and 48 h of incubation is needed for efficient growth and for typical colony morphology. After 18–24 h of incubation on blood agar plates, colonies are small (<0.5 mm) and may be irregular in shape. After 48 h, the colonies become visible (1–3 mm) and can vary in size and shape, within the same isolate or species. In gram staining, the bacteria are thin and fusiform with pointed ends (Brenner et al. 1989). Traditional methods require several days for diagnosis, but the introduction of novel methods, such as MALDI-TOF, in clinical bacteriology has significantly shortened the time to diagnosis (Zangenah et al. 2012).

Patient with congenital or acquired asplenia or with a dysfunctional spleen, alcoholics and elderly people constitute risk groups for severe *Cani* infections (Shahani and Khardori 2014; Ugai et al. 2014). However, there are only a few reports on *Cani* pathogenesis. For example, *Cani* has been shown to block the release of proinflammatory cytokines from monocytes (Shin et al. 2007). Recently, it was shown that *Cani* has a deglycosylation system that degrades exposed sugars on human IgG and on epithelial cell surfaces (Renzi et al. 2011). Finally, *Cani* has been described to be ‘serum resistant’ to 10 % New Zealand rabbit serum (Butler et al. 1985) and to normal human serum (Shin et al. 2009), but *Cani* has also been shown to be sensitive to normal human serum (Hicklin et al. 1987). Thus, the interactions between *Cani* and the immune system are not completely understood but it is possible that one or several of the described immune evasive features could contribute to the high virulence previously reported for infections with *Cani.*

In contrast to *Cani,* there is very limited knowledge on the role of human immunity in *Cyno*-infections. *Cyno* is more often reported in the case of wound infections and there is only two reports on invasive *Cyno*-infection (Khawari et al. 2005; Sarma and Mohanty 2001), whereas there are many case reports on invasive *Cani*-infections. Thus, if serum survival is a key virulence trait of *Cani*, it should be more resistant in serum than the presumably less virulent *Cyno*. To answer this question, we used a collection of clinical isolates of *Cani* and *Cyno* collected at the Karolinska University Laboratory between 2007 and 2010 and used traditional whole blood and serum bactericidal assays. The role of the complement system was dissected by the use of heat inactivation, calcium chelation and C1q-depleted sera. In addition, sera from patients deficient in mannose binding lectin (MBL-) activity were used. Finally, sera from patients with a previous history of sepsis with *Cani* were used in an attempt to elucidate pathophysiological mechanisms.

## Methods

### Reagents

Blood and hematin agar plates and phosphate buffered saline were manufactured by the Substrate Unit at Karolinska University Hospital, Stockholm, Sweden, which is accredited by SWEDAC (Swedish Board for Accreditation and Conformity Assessment).

### Collection of human serum

Venipuncture tubes without anticoagulants were used to obtain serum. Serum was aliquoted and stored in −80° until use in experiments. Sera from n = 7 healthy volunteers were pooled and constituted the “pooled serum” used in this study (ethical approval 2000/360/00, Stockholm Board of Ethics for Medical Research). The pooled serum was analyzed by Clinical Immunology, Karolinska University Laboratory, Stockholm, Sweden for complement activity and levels of immunoglobulins. Normal values were found for all analyzed parameters, including the alternative pathway (118 %), the classical pathway (97 %) and the lectin pathway (39 %), C3 (1.17 g/L), C4 (0.21 g/L) as well as IgG (12.7 g/L) and IgM (1.09 g/L). Additional sera from n = 12 additional healthy volunteers were collected and tested individually. Heat inactivated serum (HIS) was obtained by heating at 56 °C for 30 min, which inactivated the complement system. Pooled serum was treated with 10 mM EGTA-MgCl to inhibit the Ca^2^-dependent classical pathway and the lectin pathway (Abdullah et al. 2005). C1q-depleted serum was purchased from Millipore/Calbiochem, USA. The sera from mannose binding lectin (MBL) deficient patients were collected as part of another study (Bergman et al. 2012) under the ethical approval 2009/1678-31/4 (Stockholm Board of Ethics for Medical Research). Sera from patients with a previous history of *Cani*-sepsis were collected as part of the FUNGEN-study (ethical approval 2011/116-31/4, unpublished). Written informed consent was obtained from all patients.

### Bacterial strains

A total of 23 clinical strains were included in the study and they have been extensively characterized in a previous study (Zangenah et al. 2012). Eight strains of *C. canimorsus* were isolated from blood cultures and fifteen strains of *C. cynodegmi* were isolated from wound specimens. In addition, the reference strains ATCC 35978 (*C. canimorsus*, blood isolate), ATCC 49045 (*C. cynodegmi*, wound isolate) were used as controls. To validate the system of serum killing we obtained two *Salmonella* strains from the LT2-background; SL3769 (rfaG-mutant, serum sensitive) and SL3770 (parent strain, serum resistant) (Roantree et al. 1977). These strains were generously provided by professor Mikael Rhen, Karolinska Institutet. All strains were kept in glycerol stocks in −80 °C freezer, thawed and cultured for 1–2 days on blood agar plates prior to experiments.

### Serum bactericidal assay and whole blood bactericidal assay

The strains of *Cani* and *Cyno* were cultured on blood agar plates and incubated in 37 °C, at 5 % CO_2_ for 1 day. The strains did not grow in liquid broth and thus bacterial colonies were harvested directly from agar plates and suspended in 3 ml phosphate-buffered saline (PBS), pH 7.4 to optical density 0.2–0.4 (determined by A600), which corresponded to approximately 1 × 10^7^ to 1 × 10^8^ CFU/ml. The stock solution was further diluted for the various experiments.

The bactericidal assay was performed in two ways: (1) end-over-end assay using 1 ml total volume in Eppendorf tubes or (2) in the 96 microtiter well format using 100 µl total volume. The large volume was used for pooled sera and the small format was used for patient material with limited amounts. Serum was diluted in PBS prior to experiments.

For whole blood bactericidal assay, freshly drawn heparinized blood from healthy volunteers was used. For the assay, 900 µl of blood and 100 µl (1–5 × 10^6^ CFU/ml) bacteria-PBS suspensions were mixed in Eppendorf tubes and incubated at 37 °C in an end-over-end tube rotator, 6 rpm for 90 min. A viable count for each tube was performed by serial dilutions and plating of 10 µl in duplicates. *Cani* and *Cyno* were plated on blood agar plates and incubated in 37 °C, at 5 % CO_2_ for at least 2 days.

## Results

### Human serum kills *Cani* and Cyno in a time and concentration-dependent way

**Fig. 1:**
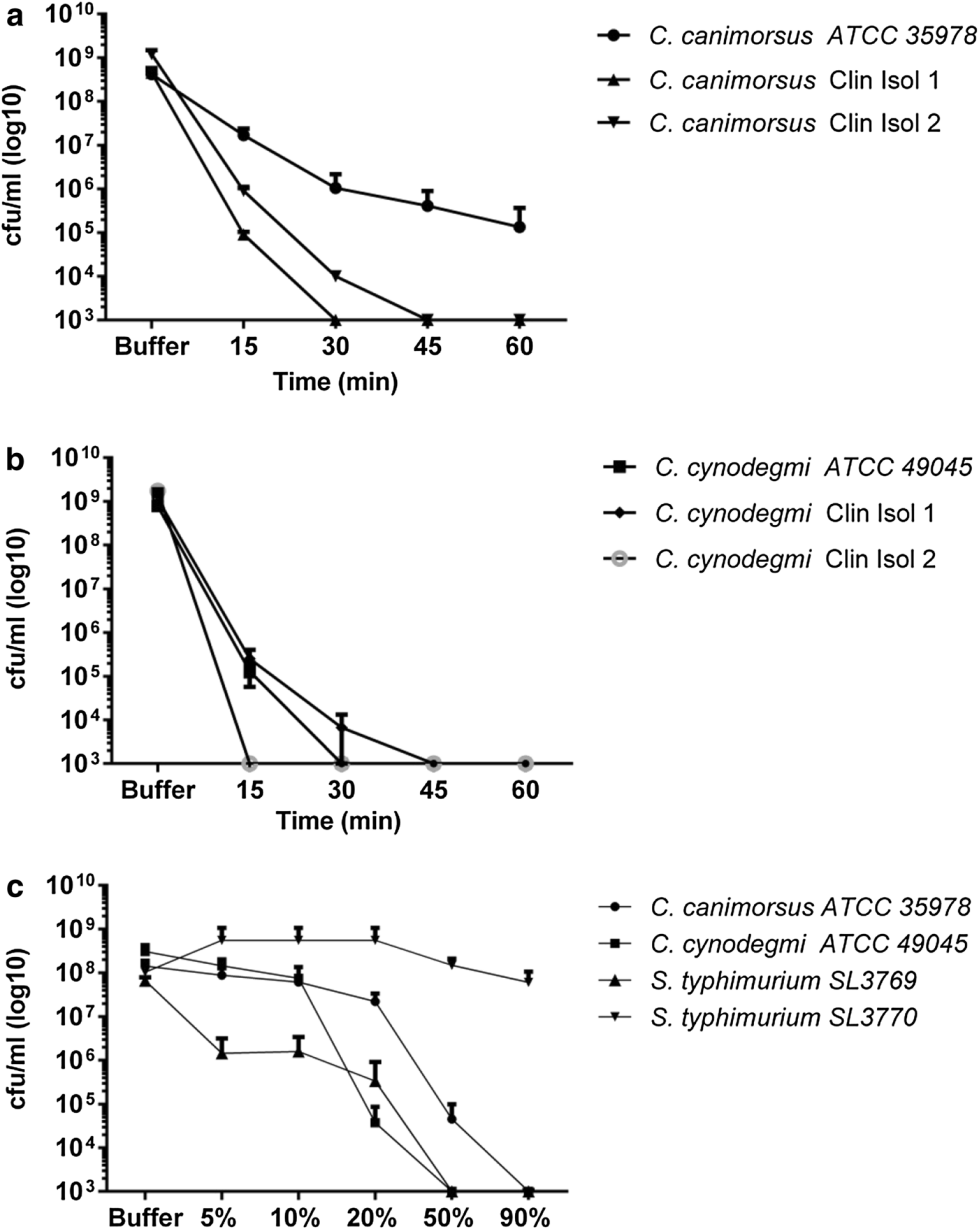
**a**, **b** Time-dependent bactericidal effect of serum against Cani and Cyno (90 % normal human serum was used). **c** Concentration-dependent bactericidal effect of normal human serum (NHS) against Cani, Cyno, the serum-sensitive Salmonella strain SL3769 and the serum-resistant Salmonella strain SL3770. Experiments were performed three times in duplicate; incubation time 3 h, the lowest level of detection was 1000 CFU/ml. *Error bars* designate standard error of the mean (SEM)

To test the hypothesis that Cani would survive better in human serum than Cyno, the ATCC reference strains and clinical isolates of Cani and Cyno were studied in a classical serum killing assay. Notably, all Cani- and Cyno-strains were rapidly killed by normal human serum from a healthy volunteer (90 % serum concentration, Fig. 1a, b). Already after 15 min, a distinct reduction was observed for both Cani and Cyno (1–3 log reduction for the Cani isolates and 4–6 log reduction for the Cyno isolates). Notably, after 60 min, no visible growth could be detected, except for the Cani reference strain, where a 3 log reduction was recorded (lowest level of detection 1000 CFU/ml, Fig. 1a, b). Surviving colonies were retested in the same serum killing assay and a complete reduction of bacterial growth was observed, thus ruling out the selection of serum resistant mutants (data not shown).

Next, we tested different concentrations of human serum (5–90 %). A small reduction of bacterial counts occurred at 5 and 10 % serum concentrations, whereas a significant killing effect was observed at 20, 50 and 90 % serum concentrations (Fig. 1c). As controls for the system the Salmonella strain SL3769, known to be ‘serum sensitive’ and Salmonella strain SL3770, known to be ‘serum resistant’ were used (Roantree et al. 1977). Importantly, strain SL3770 was not killed by serum at any concentration tested, whereas strain SL3769 was rapidly killed by human serum. Notably, Cani and Cyno appeared to follow the killing pattern of SL3769, thus supporting the notion that Cani and Cyno are susceptible to the bactericidal activity of human serum (Fig. 1c).

### Clinical isolates of Cani and Cyno are killed by human whole blood and serum

To rule out the possibility that the ATCC reference strains were different than clinical strains, we also investigated our collection of clinical isolates of Cani and Cyno (described in Zangenah et al. 2012). Most strains were readily killed by human whole blood and serum in a similar way as described for the ATCC-strains (~3 log reduction). Notably, there was some minor variation and a few strains produced viable colonies after 90 min of incubation together with human serum or whole blood (2.94–2.99 log reduction). Thus, there was some variability in the fraction of surviving bacteria in different clinical isolates. However, since the reduction of most strains was close to 3 log reduction (from an inocula of 10^6^ CFU/ml), we conclude that both the reference-strains (n = 2) and a collection of clinical isolates (n = 23) are susceptible to human serum.

### Human sera from 12 healthy volunteers are active against Cani and Cyno

To rule out inter-volunteer variation we recruited 12 healthy individuals (6 males and 6 females) who donated serum for our experiments. Importantly, sera from all individuals resulted in 2.91–3.00 log reduction of bacterial counts of Cani and Cyno, respectively.

### The magnitude of serum killing is reduced by large inocula

**Fig. 2:**
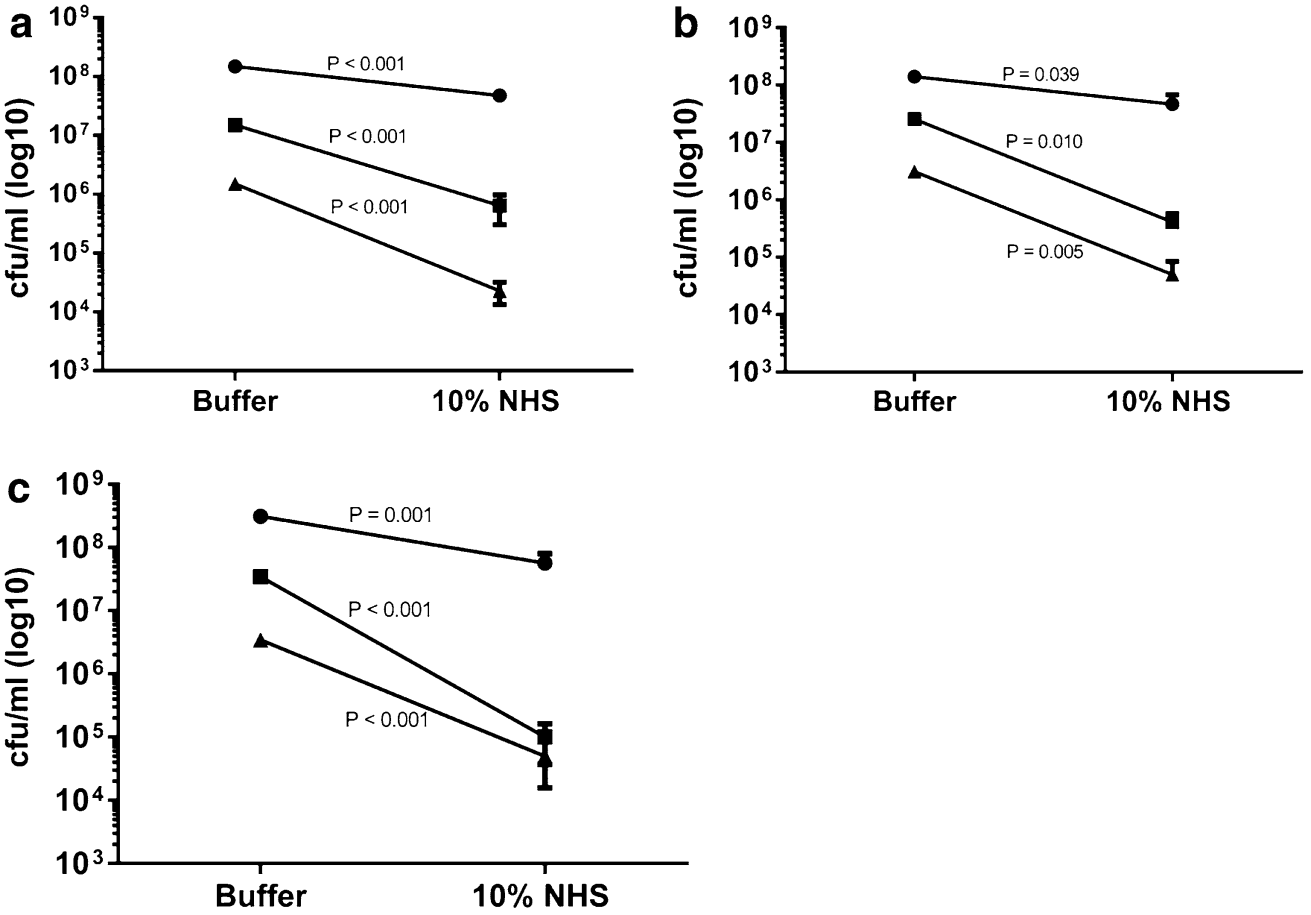
Effect of inoculum size on serum killing activity against Cani (**a**), Cyno (**b**) and a serum sensitive Salmonella strain (SL3769). The serum concentration was 10 % and the time of incubation was 180 min. Experiments were performed three times in duplicate, lowest level of detection was 1000 CFU/ml. *Error bars* designate standard error of the mean (SEM). Student’s t test was used to test for statistical significance. *NHS* normal human serum. *Circles*, *squares* and *triangles* designate large, medium and low inocula, respectively

Next, we tested the role of various inocula in the serum killing assay. To mimic previous reports where Cani has been found to be serum resistant, we used 10 % serum concentration and 180 min of incubation (Shin et al. 2009). The magnitude of the serum killing effect was significantly reduced when large inocula (10^8^–10^9^ CFU/ml, circles) were used, whereas a 1–2 log reduction was observed for lower inocula (Fig. 2a–c, squares and triangles).

### Serum killing of Cani and Cyno is mediated via the classical pathway of the complement system

**Fig. 3:**
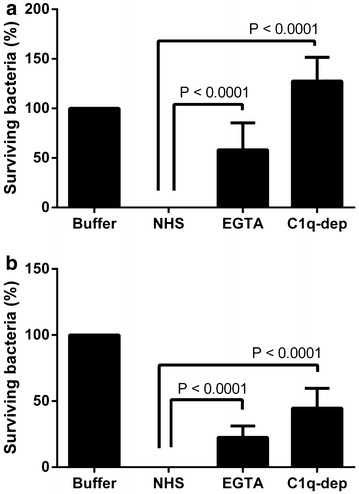
Effect of EGTA-treatment and C1q-depleted serum on bactericidal activity against Cani (**a**) and Cyno (**b**). Experiments were performed 3 times in duplicate, the serum concentration was 20 %, lowest level of detection 1000 cfu and the time of incubation was 90 min. *Error bars* designate the standard error of the mean (SEM). Student’s t test was used to test for statistical significance. *NHS* normal human serum

To study whether complement was involved in the killing of Cani and Cyno, human serum was heat inactivated in 56 °C for 30 min. Notably, this treatment completely abrogated the killing effect against both Cani and Cyno, suggesting complement dependent killing mechanisms (data not shown). To further dissect the killing mechanism of human serum we used EGTA, which is often used to block the classical pathway of the complement system (Kochi and Johnson 1988). Indeed, this treatment completely inactivated the classical (0 %) and the lectin pathways (0 %), whereas the alternative pathway was unaffected. Consequently, we added EGTA to the sera, which resulted in a significantly impaired killing effect against Cani (~60 % surviving bacteria) and Cyno (~25 % surviving bacteria). Notably, EGTA-treatment affected the killing of Cani to a larger extent than for Cyno, suggesting that Cani and Cyno are killed via different complement mediated pathways (Fig. 3).

To further study the role of the classical pathway, we obtained C1q-depleted serum. This serum was not effective against Cani and exhibited an impaired killing capacity against Cyno (Fig. 3). In fact, increased growth was observed for Cani (~125 % surviving bacteria), whereas a significant killing activity against Cyno still was evident (~40 % surviving bacteria). This was in contrast to EGTA-treatment, which resulted in a significant reduction of bacterial growth for both Cani and Cyno (Fig. 3).

**Fig. 4:**
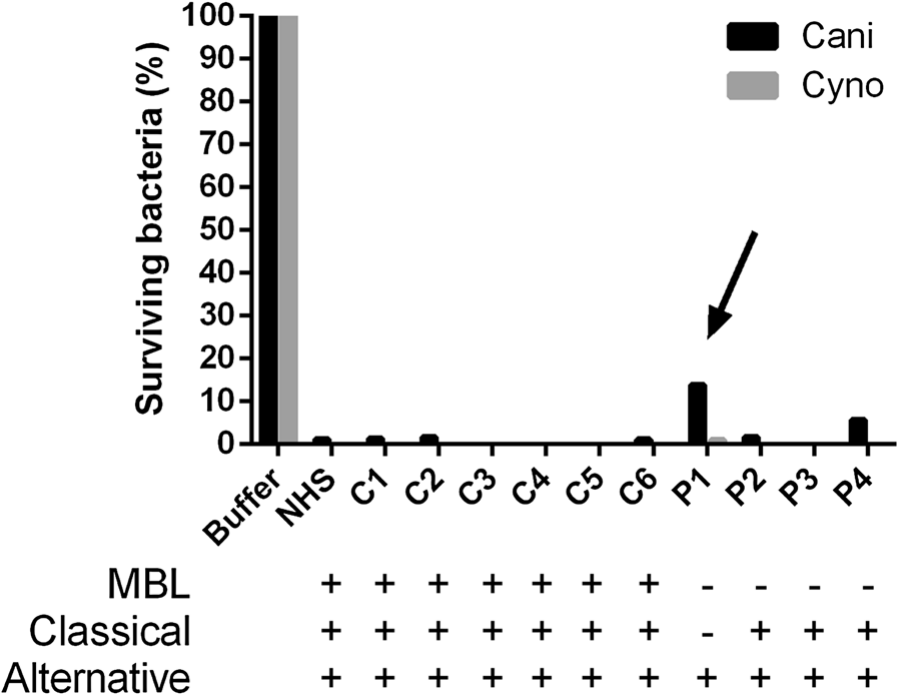
Serum of MBL-deficient patients (P1–P4) compared with IgG-deficient patients (normal MBL activity, C1–C6) and normal human serum (*NHS* pooled from healthy controls). All sera exhibited significant killing activity against Cani and Cyno, except patient P1 (*arrow*), which in addition to MBL-deficiency, had a reduced activity in the classical pathway. Inoculum was 1 × 10^6^ CFU/ml, the lowest level of detection was 1000 CFU/ml, the serum concentration was 50 % and the time of incubation was 90 min

To specifically investigate the involvement of the lectin pathway, we used sera from patients with mannose binding lectin (MBL) deficiency (0–3 %, reference 100 %). All patients exhibited potent killing activity against Cani and Cyno (Fig. 4). Notably, one patient had a slightly impaired killing capacity, but in addition to MBL-deficiency this patient had a decreased function in the classical pathway (3 %) as determined by the clinical laboratory (Fig. 4, arrow). Combined with results from EGTA/C1q-experiments, these data suggest that the classical pathway contributes significantly to the killing of Cani and that Cyno is killed via partly different mechanisms, possibly via the alternative complement pathway.

### Sera from patients with a previous history of Cani-sepsis have intact serum bactericidal activity and normal levels of immunoglobulins but have signs of MBL-deficiency

Finally, whole blood and sera from patients with a recent history of sepsis with Cani was tested against the autologous Cani-strain (the strain originally isolated from the patient’s blood) and against another clinical Cani-isolate. Notably, all 3 patients exhibited full bactericidal activity for whole blood and serum against both the autologous strain and against the other clinical Cani-isolate. In fact, no viable bacteria were present after 90 min incubation in 20 % serum (~5 log reduction for all strains, lowest level of detection 10^3^ CFU/ml, data not shown). A detailed clinical and immunological workup was performed for these patients (Table 1). There was no obvious immunodeficiency present that could explain why these particular individuals experienced a severe Cani-infection. However, they were above 50 years of age, which have been described as a risk factor in previous reports (Lion et al. 1996). One patient had a possible overconsumption of alcohol, and another patient was on iron supplementation; both circumstances are considered as risk factors for Cani-infections (Lion et al. 1996). In addition, 2 out of 3 patients were deficient in the MBL-pathway, which was interesting but did not explain their infectious susceptibility against Cani (Table 1).

**Table 1:** Clinical history for the patients with a history of sepsis with *C. canimorsus.* MBL. mannose binding lectin

	Patient 1	Patient 2	Patient 3
Age	60	66	72
Sex	M	M	F
Animal	Dog	Dog	Dog
Type of contact	Minor wound	Minor wound	Minor wound
Wound-infection	No	No	No
Sepsis	Yes	Yes	Yes
Time to hospital	36 h	Unknown	3 days
Clinical history	Age. Alcohol	Age. Iron supplementation	Age. Smoking
Sequelae	No	Balance-problems, fatigue	Fatigue
IgG	12.2 g/l	5.82 g/l	9.2 g/l
IgA	3.36 g/l	1.09 g/l	2.76 g/l
IgM	0.98 g/l	1.8 g/l	0.82 g/l
Classical	103 %	95 %	107 %
Alternative	95 %	66 %	115 %
MBL	0 %	1 %	100 %

## Discussion

### Statement of principal findings

Here we show that Cani and Cyno are readily killed by human serum in a time and concentration dependent way. This effect was dependent on complement activity since heat inactivation, EGTA and C1q-depletion impaired killing. Interestingly, Cani was more dependent on the classical pathway compared with Cyno, suggesting a possibly differential effect of the complement system against these bacteria. Further, experiments on patients with MBL-deficiency did not support a role for the lectin pathway in the killing of Cani and Cyno. Finally, we investigated sera from three patients with a history of sepsis with Cani and found their sera to have normal complement activity as well as normal killing capacity against Cani.

Importantly, our observations are in contrast to studies in the literature where Cani has been shown to be completely resistant to human serum (Shin et al. 2009). The mechanism of serum resistance has been attributed to a putative glycosyltransferase, which affects the LPS-structure (Shin et al. 2009). Since our results are different from previous data we have tried to understand the reason for this discrepancy in detail.

### There are several reasons why our results differ from previous reports

First, we have studied a collection of clinical isolates collected directly from clinical specimens of blood and wounds, respectively. The collection contains 23 strains and is extensively characterized with regards to diagnostic aspects (biochemistry, 16S rRNA sequencing and MALDI-TOF) (Zangenah et al. 2012). In addition, we have performed whole genome sequencing of all isolates, which have confirmed their identity and phylogeny (Zangenah et al., unpublished). Second, we have elaborated the serum bactericidal assay procedure in detail, including dependence of time, serum concentration and inoculum size. In fact, we show that there is a possible saturation effect with very large inocula (10^8^–10^9^ CFU/ml). This is an unusual finding since it has been shown for E. coli that a high inoculum (10^10^ CFU) was equally well killed as a 6 log lower inoculum (10^4^ CFU) (Olling 1977). It is possible that this saturation effect could contribute to the observation of serum resistance in previous papers, where high inocula (10^7^ and 10^8^ CFU/ml) have been used (Shin et al. 2007, 2009). Another aspect is the role of serum concentration in serum bactericidal assays. It has been suggested that too low serum concentration depletes the complement system of key components that may be diluted, i.e. the stoichiometry of key complement factors can be disturbed if too low concentrations are used, with a potential risk of having false negative results (Schreiber et al. 1979). In fact, the alternative pathway was disturbed when human serum was diluted 1:16 (Schreiber et al. 1979) and the activity of guinea pig serum was abrogated when used in concentrations lower than 10 % (Clas and Loos 1980). We have used 20 % serum for most of the experiments, and 10 % for some parts of the study. Previous reports have mainly used 10 % serum, which possibly could have played a role in previous observations of ‘the serum resistant’ phenotype. In fact, our results indicate that 10 % may be too diluted serum to exert significant killing against Cani and Cyno (Fig. 1c). However, at the more physiological concentration of 20 % serum there is a significant reduction of both Cani and Cyno, which suggest that both these species are indeed to be described as ‘serum sensitive’. At least, it is fair to state that Cani and Cyno are significantly more sensitive to serum than truly “serum resistant strains”, such as Salmonella SL3770 (Fig. 1c) and Streptococcus pyogenes (data not shown). Finally, we have studied n = 12 different individuals to evaluate a possible role of inter-individual variation and all sera tested resulted in 2.94–3 log reduction of all tested strains. Even though not complete killing was observed for all strains or sera tested, our data do not support the notion that Cani and Cyno should be described as ‘serum resistant’ bacteria.

### Nevertheless, several concerns against our results may be raised

First, could our assay conditions have rendered the bacteria falsely serum sensitive? We do acknowledge that the assay conditions are crucial for correct results. Also in our hands the results varied depending on which method that was used. Importantly, the methods used here (end-over-end rotation or 96 well microtiter plate) gave robust and reproducible results, again corroborating the important role of serum in the killing of these bacteria. Second, we have used the well described Salmonella strain SL3770, which is known to be serum resistant. This strain was completely resistant to serum mediated killing up to 90 % serum concentration (Fig. 1c). Importantly, both Cani and Cyno followed approximately the same killing kinetics as the serum susceptible Salmonella strain SL3769, which further indicates that Cani and Cyno should be considered as ‘serum-sensitive’. The Salmonella strain SL3769 has a mutation in a glycosyl transferase encoded by the gene rfaG, which lead to increased susceptibility to a number of antibiotics and to killing by human serum (Roantree et al. 1977). Finally, an additional problem could be that bacteria were scraped off the plates and not grown to mid logarithmic phase prior to experiments. This is a valid point of criticism since mid-logarithmic phase bacteria are considered to be a gold standard in antimicrobial assays. Bacteria that are scraped off from plates constitute a mixture between growing, dead and dormant bacteria (stationary phase) and are thus not perfect for use in antimicrobial assays. However, Cani and Cyno are very difficult to grow in broth and we could not obtain mid log phase broths for our experiments. Therefore, we used the second best approach, i.e. scraping bacteria off plates, which probably have the net effect that bacteria appear to be less resistant against various antimicrobial agents, including complement deposition. Dividing bacteria in mid log phase are considered to be more susceptible to killing by both antibiotics (Lewis 2007) and antimicrobial peptides (Kristian et al. 2007). This is important since it has been shown for E. coli that susceptible strains can appear to be completely refractory to serum bactericidal effects if bacteria are harvested before the onset of the exponential phase (Davis and Wedgwood 1965). Thus, it is possible that such circumstances could be part of the explanation for why previous reports have concluded that Cani has a serum resistant phenotype.

### What do these results mean?

We can conclude that Cani and Cyno indeed are sensitive to human serum. This is potentially important since it is well described that individuals with various degrees of immunodeficiency are more susceptible to infections with Cani (Lion et al. 1996). For example, patients with splenectomy, alcoholism, complement deficiency or various cancers have been described with severe forms of Cani-infections. However, the precise mechanisms behind this increased susceptibility have not been clarified. In addition, approximately half of patients with invasive Cani-infection have no defined immunosuppressive disorder. Here we had the possibility to perform clinical and laboratory investigations of three patients with a history of Cani sepsis. The clinical picture was similar: a contact with the house hold dog resulted in an innocuous wound, for which they did not seek medical assistance initially. However, after 2–3 days a sudden onset of fever, chills and malaise occurred. This resulted in medical emergency visits, sometimes requiring an ambulance. In the emergency room, ‘sepsis of unknown cause’ was diagnosed and empirical treatment with intravenous cephalosporins resulted in a rapid recovery. After 5–7 days, an answer came from the clinical bacteriology department with a positive blood culture of Cani. All had normal activity of the classical and alternative complement pathways, but 2/3 patients had a deficiency in the MBL-pathway. Notably, all 3 patients’ sera killed their autologous strain of Cani. Thus, the explanation for their susceptibility to this bacterium has not been clarified but an involvement of opsonization and uptake of phagocytic cells cannot be ruled out.

### Unanswered questions and future research

Combined, our results imply that whole blood and serum rapidly kills these bacteria. Since our approach includes many clinical strains, a large number of healthy volunteers, patients with MBL-deficiency as well as patients with a history of Cani-sepsis, we are confident to conclude that both Cani and Cyno are indeed sensitive to human whole blood and serum.

The current results could be important for the increasing group of immunocompromised patients in hospitals and society. Notably, in Sweden there are 1 million dogs and many patients spend time with their house hold dogs after cancer treatment or transplantation. It is well known that the complement system may be impaired after transplantation, which could have a profound impact on these types of infections. Thus, a detailed understanding of immunity against Cani and Cyno could direct therapeutic or prophylactic strategies in the immunocompromised host, including transplanted or cancer patients.

## References

[CR1] Abdullah M, Nepluev I, Afonina G, Ram S, Rice P, Cade W, Elkins C (2005). Killing of dsrA mutants of Haemophilus ducreyi by normal human serum occurs via the classical complement pathway and is initiated by immunoglobulin M binding. Infect Immun.

[CR2] Bergman P, Norlin AC, Hansen S, Rekha RS, Agerberth B, Björkhem-Bergman L, Ekström L, Lindh JD, Andersson J (2012). Vitamin D3 supplementation in patients with frequent respiratory tract infections: a randomised and double-blind intervention study. BMJ Open.

[CR3] Bobo RA, Newton EJ (1976). A previously undescribed gram-negative bacillus causing septicemia and meningitis. Am J Clin Pathol.

[CR4] Brenner DJ, Hollis DG, Fanning GR, Weaver RE (1989). *Capnocytophaga canimorsus* sp. nov. (formerly CDC group DF-2), a cause of septicemia following dog bite, and *C. cynodegmi* sp. nov., a cause of localized wound infection following dog bite. J Clin Microbiol.

[CR5] Butler T (2015). * Capnocytophaga canimorsus*: an emerging cause of sepsis, meningitis, and post-splenectomy infection after dog bites. Eur J Clin Microbiol Infect Dis Off Publ Eur Soc Clin Microbiol.

[CR6] Butler T, Johnston KH, Gutierrez Y, Aikawa M, Cardaman R (1985). Enhancement of experimental bacteremia and endocarditis caused by dysgonic fermenter (DF-2) bacterium after treatment with methylprednisolone and after splenectomy. Infect Immun.

[CR7] Clas F, Loos M (1980). Killing of the S and Re forms of Salmonella minnesota via the classical pathway of complement activation in guinea-pig and human sera. Immunology.

[CR8] Davis SD, Wedgwood RJ (1965). Kinetics of the Bactericidal Action of Normal Serum on Gram-Negative Bacteria. J Immunol.

[CR9] Gasch O, Fernandez N, Armisen A, Verdaguer R, Fernandez P (2009). Community-acquired * Capnocytophaga canimorsus* meningitis in adults: report of one case with a subacute course and deafness, and literature review. Enferm Infecc Microbiol Clin.

[CR10] Hicklin H, Verghese A, Alvarez S (1987). Dysgonic fermenter 2 septicemia. Rev Infect Dis.

[CR11] Khawari AA, Myers JW, Ferguson DA, Moorman JP (2005). Sepsis and meningitis due to Capnocytophaga cynodegmi after splenectomy. Clin Infect Dis Off Publ Infect Dis Soc Am.

[CR12] Kochi SK, Johnson RC (1988). Role of immunoglobulin G in killing of Borrelia burgdorferi by the classical complement pathway. Infect Immun.

[CR13] Kristian SA, Timmer AM, Liu GY, Lauth X, Sal-Man N, Rosenfeld Y, Shai Y, Gallo RL, Nizet V (2007). Impairment of innate immune killing mechanisms by bacteriostatic antibiotics. FASEB J Off Publ Fed Am Soc Exp Biol.

[CR14] Lewis K (2007). Persister cells, dormancy and infectious disease. Nat Rev Microbiol.

[CR15] Lion C, Escande F, Burdin JC (1996). * Capnocytophaga canimorsus* infections in human: review of the literature and cases report. Eur J Epidemiol.

[CR16] Oehler RL, Velez AP, Mizrachi M, Lamarche J, Gompf S (2009). Bite-related and septic syndromes caused by cats and dogs. Lancet Infect Dis.

[CR17] Olling S (1977). Sensitivity of gram-negative bacilli to the serum bactericidal activity: a marker of the host-parasite relationship in acute and persisting infections. Scand J Infect Dis Suppl.

[CR18] Renzi F, Manfredi P, Mally M, Moes S, Jeno P, Cornelis GR (2011). The N-glycan glycoprotein deglycosylation complex (Gpd) from * Capnocytophaga canimorsus* deglycosylates human IgG. PLoS Pathog.

[CR19] Roantree RJ, Kuo TT, MacPhee DG (1977). The effect of defined lipopolysaccharide core defects upon antibiotic resistances of Salmonella typhimurium. J Gen Microbiol.

[CR20] Sarma PS, Mohanty S (2001). Capnocytophaga cynodegmi cellulitis, bacteremia, and pneumonitis in a diabetic man. J Clin Microbiol.

[CR21] Schreiber RD, Morrison DC, Podack ER, Muller-Eberhard HJ (1979). Bactericidal activity of the alternative complement pathway generated from 11 isolated plasma proteins. J Exp Med.

[CR22] Shahani L, Khardori N (2014). Overwhelming *Capnocytophaga canimorsus* infection in a patient with asplenia. BMJ Case Rep.

[CR23] Shin H, Mally M, Kuhn M, Paroz C, Cornelis GR (2007). Escape from immune surveillance by * Capnocytophaga canimorsus*. J Infect Dis.

[CR24] Shin H, Mally M, Meyer S, Fiechter C, Paroz C, Zaehringer U, Cornelis GR (2009). Resistance of * Capnocytophaga canimorsus* to killing by human complement and polymorphonuclear leukocytes. Infect Immun.

[CR25] Suzuki M, Kimura M, Imaoka K, Yamada A (2010). Prevalence of * Capnocytophaga canimorsus* and *Capnocytophaga cynodegmi* in dogs and cats determined by using a newly established species-specific PCR. Vet Microbiol.

[CR26] Ugai T, Sugihara H, Nishida Y, Yamakura M, Takeuchi M, Matsue K (2014). * Capnocytophaga canimorsus* sepsis following BMT in a patient with AML: possible association with functional asplenia. Bone Marrow Transpl.

[CR27] Zangenah S, Ozenci V, Borang S, Bergman P (2012). Identification of blood and wound isolates of *C. canimorsus* and *C. cynodegmi* using VITEK2 and MALDI-TOF. Eur J Clin Microbiol Infect Dis Off Publ Eur Soc Clin Microbiol.

